# Non-invasive Quantification of Fat Deposits in Skeletal Muscle Predicts Cardiovascular Outcome in Kidney Failure

**DOI:** 10.3389/fphys.2020.00130

**Published:** 2020-02-25

**Authors:** Mehdi Keddar, Thibaut Muylle, Emmanuelle Carrie, Pierre Trefois, Maxime Nachit, Ralph Crott, Claudine Christiaens, Bert Bammens, Michel Jadoul, Eric Goffin, Johann Morelle

**Affiliations:** ^1^Division of Nephrology, Cliniques Universitaires Saint-Luc, Brussels, Belgium; ^2^Institut de Recherche Expérimentale et Clinique, UCLouvain, Brussels, Belgium; ^3^Department of Medical Imaging, Cliniques Universitaires Saint-Luc, Brussels, Belgium; ^4^Department of Imaging and Pathology, KU Leuven, Leuven, Belgium; ^5^Consultant, Colombiers, France; ^6^Department of Nephrology and Renal Transplantation, University Hospitals Leuven, Leuven, Belgium; ^7^Department of Microbiology and Immunology, Nephrology and Renal Transplantation Research Group, KU Leuven, Leuven, Belgium

**Keywords:** cardiovascular disease, myosteatosis, chronic kidney disease, end-stage kidney disease, peritoneal dialysis

## Abstract

Fat accumulation in skeletal muscle was recently established as a major risk factor for cardiovascular disease (CVD) in the general population, but its relevance for patients with kidney failure is unknown. Here we examined the potential association between muscle radiation attenuation (MRA), a non-invasive indicator of fat deposits in muscle, and cardiovascular events in patients with kidney failure treated with peritoneal dialysis (PD) and investigated dynamic changes and determinants of MRA in this population. We retrospectively assessed MRA on computed tomography images collected yearly in 101 incident patients with kidney failure starting PD between January 2006 and December 2015. After a median of 21 months on dialysis, 34 patients had 58 non-fatal cardiovascular events, and 22 patients had died. Baseline MRA was associated with cardiovascular events during time on dialysis, and patients with higher MRA (reflecting lower amounts of fat in muscle) showed a reduced incidence of CVD, independently of traditional risk factors (adjusted HR, 0.91; 95% CI, 0.86–0.97, *P* = 0.006). Multivariate regression analysis identified old age, female gender, visceral fat area, and low residual urine volume as independent determinants of MRA. As compared with reference values from a healthy population, patients with kidney failure had lower MRA (i.e., increased fat accumulation), independently of age, gender, and body-mass index. The subset of patients who underwent kidney transplantation showed a significant increase in MRA after restoration of kidney function. These observations expand the association between ectopic fat accumulation and CVD to the population on dialysis, and suggest that kidney failure is reversibly associated with fatty muscle infiltration.

## Introduction

Chronic kidney disease (CKD) dramatically increases the risk of cardiovascular disease (CVD), which culminates in patients with kidney failure in a 10- to 20-fold increased hazard as compared with age- and gender-matched controls (Go et al., 2004; Gansevoort et al., 2013). Among patients with end-stage kidney disease (ESKD), CVD is the leading cause of death and a major source of morbidity, disabilities, and hospitalizations (Gansevoort et al., 2013). The unacceptably high CVD risk in this population is related to a high prevalence of traditional risk factors, such as hypertension and diabetes, but also to non-traditional, kidney-specific mechanisms (Gansevoort et al., 2013). Among the latter, metabolic disturbances represent an established feature of CKD, and are independently associated with CVD morbidity and mortality in patients with ESKD (DeFronzo et al., 1981; de Boer et al., 2012, 2016; Spoto et al., 2016). It has been suggested that differences in body composition and adiposity might explain, at least in part, the altered glucose and insulin homeostasis associated with moderate to severe CKD (de Boer et al., 2016).

Ectopic fat accumulation, which refers to an increased amount of lipids around and within non-adipose tissue organs such as the skeletal muscle, liver, and heart, is now recognized as a major risk factor for metabolic alterations and CVD. It results from either an inappropriate and sustained positive energy balance, leading to the spill over of energy storage from subcutaneous adipose tissue; from impaired storage of energy in fat deposits (e.g., in congenital or acquired lipodystrophies); or from defects in adipocyte metabolism (Shulman, 2014; Samuel and Shulman, 2016; Petersen and Shulman, 2018). The development of highly sensitive imaging-based methods to quantify fat distribution, and their application to large cohorts of non-CKD individuals uncovered a strong association between ectopic fat accumulation and insulin resistance, atherogenic dyslipidemia, hypertension, and CVD (Neeland et al., 2018, 2019). In particular, decreased muscle radiation attenuation (MRA) on computed tomography reflects excessive fat deposition in the tissue and is associated with poor cardiovascular outcome (Brumbaugh et al., 2012; Therkelsen et al., 2013; Lim and Meigs, 2014; Miljkovic et al., 2015; Zhao et al., 2016; Sugai et al., 2018).

To date, no study investigated the association between fat distribution and clinical outcomes among patients with ESKD, who are at risk for both metabolic alterations and CVD. We therefore tested the hypothesis that non-invasive quantification of fat accumulation in muscle may help identifying ESKD patients at risk for CVD. We also investigated dynamic changes and determinants of MRA in this population.

## Materials and Methods

### Study Participants, Data Collection, and Outcomes

The cohort included all consecutive adult ESKD individuals starting peritoneal dialysis (PD) between January 2006 and December 2015 at Saint-Luc Academic Hospital, Brussels, Belgium. Patients were followed during time on PD, until death, kidney transplantation, transfer to hemodialysis, or end of study on 31 December 2017. Only four out of 101 patients enrolled in this study were lost to follow-up. None of the relevant baseline characteristics differed between the four patients who were lost to follow-up from those who remained in the study. Time-independent variables were collected for each patient at study entry. Time-dependent variables, including parameters of fat distribution and body composition, were collected at dialysis initiation then yearly.

The primary outcome was the occurrence of at least one non-fatal CVD event during time on dialysis, including myocardial infarction or coronary revascularization; ischemic stroke, transient ischemic attack, or cerebrovascular revascularization; lower limb necrosis or peripheral arterial revascularization (Nguyen et al., 2010). Residual kidney function (RKF) was estimated from daily urine volume, a robust indicator of outcome in the dialysis population (Shemin et al., 2001; Shafi et al., 2010; Vilar and Farrington, 2011). Glucose and icodextrin exposures were quantified as previously described (Morelle et al., 2015).

The study was in accordance with the World Medical Association’s Declaration of Helsinki, the Belgian law related to experiments in humans dated 7 May 2004, the General Data Protection Regulation 2016/679 and the Belgian law of 30 July 2018 regarding the protection of personal data. The Ethical Review Board of Cliniques universitaires Saint-Luc and UCLouvain approved the study (approval number: 2015/10DEC/675) and waived the requirement to obtain informed consent based on the observational and retrospective design of the study. Patients for whom individual data are shown have provided written informed consent to the use of anonymized pictures or results of biological analyses.

### Computed Tomography Scanning and Evaluation of Body Composition

Body composition was assessed by secondary analysis of electronically stored CT images, obtained at dialysis start, then yearly in every patient, as part of routine clinical practice. The goal of the procedure, initiated in January 2006 for all patients starting PD, was to identify patients at risk for or with early encapsulating peritoneal sclerosis, a dramatic complication of PD (Morelle et al., 2015). At the time this procedure was initiated, the limited yield of CT scan to identify patients at risk for severe peritoneal fibrosis was not established (Brown et al., 2017). Importantly, all CT scans were non-contrast enhanced, as iodine-contrast media interferes with MRA measurement (Boutin et al., 2016).

The third lumbar vertebra (L3) was chosen as a standard landmark to assess parameters of body composition and fat distribution, as previously described (Loumaye et al., 2017). Skeletal muscle and adipose tissue were identified by the use of Hounsfield unit (HU) thresholds –29 to + 150 and −190 to −30 HUs, respectively, and quantified by the Slice-O-Matic software, version 4.3 (Tomovision, Montreal, Canada), as previously described (Prado et al., 2008; Martin et al., 2013; Loumaye et al., 2017). Skeletal muscle index (SMI) was calculated as the total skeletal muscle area normalized for stature—as is conventional for body composition components (cm^2^/m^2^) (Prado et al., 2008; Loumaye et al., 2017). Mean MRA (HU) was reported for the entire muscle area at the third lumbar vertebra (Martin et al., 2013). Values of muscle density of 31–100 HU, 0–30 HU, and −29–0 were previously defined as normal (a value defined as two standard deviations below the mean attenuation value of muscles of young healthy persons), low, or abnormal, respectively (Goodpaster et al., 2000a, b). A single trained observer, blind to patient’s status, analyzed all images. Reference values from a healthy population were derived from a previously reported study which applied the Slice-O-Matic software on non-contrast abdominal computed tomography images from 420 Caucasian, medically approved living kidney donor candidates (van der Werf et al., 2018).

### Serum Levels of Uremic Retention Solutes

Total serum levels of uremic retention solutes were assessed on serum using a highly sensitive ultra-performance liquid chromatography–tandem mass spectrometry method, as previously described (de Loor et al., 2016). Briefly, sample preparation was performed with an Ostro pass-through protocol (Waters, Zellik, Belgium). Chromatographic separation was achieved on an Acquity CSHFluoroPhenyl column (50 × 2.5 mm; 1.7 μm particle size; Waters, Zellik, Belgium). The mobile phase was a gradient of 0.1% formic acid in Milli-Q water and pure methanol at a flow rate of 0.5 ml min^–1^. Total run time was 8 min. Detection was performed using a Xevo TQS tandem mass spectrometer (Waters, Zellik, Belgium), with alternated positive and negative electrospray ionization.

### Statistical Analyses

Results are presented as means ± SD or median [interquartile range (IQR)] for continuous variables and as numbers and proportions for categorical variables. Age was defined at start of PD; hypertension, diabetes, and history of coronary heart disease were constructed as binary presence/absence variables. Continuous variables were expressed in their natural units without standardization. Univariate comparisons between the CVD and non-CVD groups were performed using unpaired *t*-test, or χ^2^ test, as appropriate. Time to event analyses were performed by Cox regression and competing risk survival analyses, using the Fine and Gray method to correct for competing risk outcomes. For multivariable analyses, the following pre-specified covariates were included in the model: age at dialysis start, HDL-cholesterol level, presence of hypertension and diabetes, history of coronary heart disease, body-mass index, residual urine volume, plasma level of high-sensitivity C-reactive protein, and MRA. Collinearity between variables was quantified using variance inflation factors and condition numbers. Variance inflation factors > 10 or condition numbers > 30 suggested excessive correlation between variables. Because collinearity diagnostics showed high condition numbers due to significant correlations between age and MRA, sensitivity analyses were performed after exclusion of the variable age. Although MRA also modestly correlated with body-mass index, condition numbers were less than 30 and, therefore, body-mass index was included in all analyses. The likelihood ratio test was also used in nested models to determine whether exclusion of variable age or body-mass index altered model performance as compared with a model with MRA alone. To avoid overfitting, we limited the number of explanatory variables to achieve a ratio of at least 20 observations per coefficient whenever possible, and validated models using shrinkage statistics (Bilger and Manning, 2015). Linear regression analysis using a random intercept mixed model was used to evaluate the determinants of MRA, and the effect of time on dialysis on the parameters of body composition. Time on PD was defined as a yearly discrete period in the linear regression models. A Wilcoxon matched-pairs signed rank test was used to compare estimated glomerular filtration rate and MRA before and after transplantation in a small subset of patients. All statistical analyses were performed using GraphPad Prism (version 7.0) or Stata (version 15.0) software. All tests were two-tailed and a *P*-value < 0.05 was considered significant.

## Results

### Patients and Outcomes

The cohort (Table 1) included 101 patients, mostly of Caucasian origin, aged (±SD) 56 (±18) years, with a male-to-female ratio of 2.0, and a body mass index of 25.3 (±4.7) kg/m^2^. Thirty-one percent had diabetes; 81%, hypertension; 12%, coronary heart disease; and 96%, RKF at dialysis start, with a mean daily urine output of 1539 (±695) ml. Eighty-five percent received angiotensin converting enzyme inhibitors or angiotensin receptor blockers; 57%, statins; and 19% and 7%, insulin and oral antidiabetic agents, respectively. A total of 215 CT scans performed at dialysis start (median time on dialysis [IQR] of 33.5 days [6–76]) and during therapy were analyzed (Supplementary Table S1). Parameters of body composition included subcutaneous and visceral fat area (SFA and VFA), SMI, and MRA—an established surrogate for ectopic fat deposition in muscle, also termed myosteatosis (Goodpaster et al., 2000a; Prado et al., 2008; Martin et al., 2013; Loumaye et al., 2017). At baseline, mean SFA was 143 (±88) cm^2^; VFA, 118 (±86) cm^2^; and SMI 51 (±9) cm^2^/m^2^, and MRA 36 (±12) HUs (Table 1).

**TABLE 1:** Baseline characteristics.

Characteristic	Cohort *n* = 101	No CVD event *n* = 67	≥1 CVD event *n* = 34	*P-*value
Female gender—*n* (%)	34 (34)	21 (31)	13 (38)	0.5
Age at ESKD—years	56 ± 18	53 ± 19	62 ± 13	0.004
Ethnicity—*n* (%)				0.4
Caucasian	94 (93)	62 (93)	32 (94)	
Asian	3 (3)	3 (4)	0 (0)	
African	4 (4)	2 (3)	2 (6)	
Cause of ESKD—*n* (%)				0.003
Glomerulonephritis	35 (35)	28 (42)	7 (21)	
Diabetic nephropathy	19 (19)	5 (7)	14 (41)	
Interstitial nephritis	10 (10)	7 (10)	3 (9)	
Hypertension/renal vascular disease	10 (10)	6 (8)	4 (12)	
Polycystic kidney disease	6 (6)	3 (5)	3 (9)	
Miscellaneous/unknown	21 (20)	18 (27)	3 (8)	
Charlson comorbidity index	5.6 ± 2.6	5.2 ± 2.7	6.3 ± 2.3	0.03
Davies comorbidity index	1.5 ± 1.2	1.3 ± 1.1	2.1 ± 1.3	0.002
Hypertension—*n* (%)	82 (81)	50 (74)	32 (94)	0.02
Diabetes—*n* (%)	31 (31)	13 (19)	18 (53)	0.001
History of CHF—*n* (%)	7 (7)	5 (7)	2 (6)	0.8
History of CHD—*n* (%)	12 (12)	5 (7)	7 (21)	0.05
Kidney transplant waiting list—*n* (%)	60 (59)	44 (66)	16 (47)	0.07
Drug therapy				
ACEi—*n* (%)	50 (50)	29 (43)	21 (62)	0.08
ARB—*n* (%)	35 (35)	22 (33)	13 (38)	0.6
Beta-blockers—*n* (%)	39 (39)	21 (31)	18 (53)	0.04
Corticosteroids—*n* (%)	15 (15)	8 (12)	7 (21)	0.3
Statins—*n* (%)	58 (57)	33 (49)	25 (74)	0.02
Oral antidiabetic agent—*n* (%)	7 (7)	4 (6)	3 (9)	0.6
Insulin—*n* (%)	19 (19)	6 (9)	13 (38)	<0.001
Body mass index—kg/m^2^	25.3 ± 4.7	24 ± 4	27 ± 5	0.003
Systolic BP—mmHg	140 ± 25	140 ± 27	140 ± 22	0.9
Diastolic BP—mmHg	82 ± 14	82 ± 15	82 ± 12	0.9
Plasma hsCRP—mg/dL	2.2 ± 3.9	1.6 ± 2.8	3.4 ± 5.3	0.04
HDL-cholesterol—mg/dL	47 ± 16	48 ± 17	45 ± 15	0.4
Triglycerides—mg/dL	159 ± 89	165 ± 96	148 ± 77	0.4
Residual kidney function—*n* (%)	97 (96)	65 (97)	32 (94)	0.5
Residual urine volume—mL/day	1539 ± 695	1542 ± 682	1534 ± 733	0.9
Parameters of peritoneal transport				
Net UF 3.86% glucose—mL/4 h	494 ± 339	507 ± 297	464 ± 431	0.7
D/P_creat_ at 4 h	0.77 ± 0.09	0.77 ± 0.10	0.79 ± 0.08	0.5
Albumin loss—mg/4 h	1095 ± 446	1083 ± 507	1114 ± 339	0.5
Automated PD—*n* (%)	56 (55)	27 (40)	18 (53)	0.2
Fat distribution and body composition				
Subcutaneous fat area—cm^2^	143 ± 88	132 ± 72	166 ± 114	0.4
Visceral fat area—cm^2^	118 ± 86	109 ± 79	140 ± 96	0.2
Skeletal muscle index—cm^2^	51 ± 9	51 ± 9	51 ± 8	0.9
Muscle radiation attenuation—HU	36 ± 12	38 ± 12	31 ± 11	0.006

*Results are presented as mean ± SD or number (*n*) and percentage (%) as appropriate. CVD, cardiovascular disease; CHF, congestive heart failure; CHD, coronary heart disease; ACEi, angiotensin-converting-enzyme inhibitors; ARB, angiotensin II receptor blockers; BP, blood pressure; hsCRP, high-sensitivity C-reactive protein; HDL, high density lipoprotein; UF, ultrafiltration; D/P_*creat*_, dialysate-over-plasma creatinine ratio; PD, peritoneal dialysis; HU, Hounsfield units.*

Patients were followed on PD until death, transplantation, transfer to hemodialysis, or end of study period. After a median of 21 months on dialysis, 31% of patients had received a kidney transplant; 31% had been transferred to hemodialysis; 22% had died; 12% were still on PD; and 5% had been lost to follow-up or experienced recovery of kidney function. During follow-up, 34 patients presented 58 CVD events (Supplementary Table S2), including lower limb necrosis or revascularization (50%), myocardial infarction or revascularization (29%), and stroke or transient ischemic attack (21%).

**FIGURE 1:**
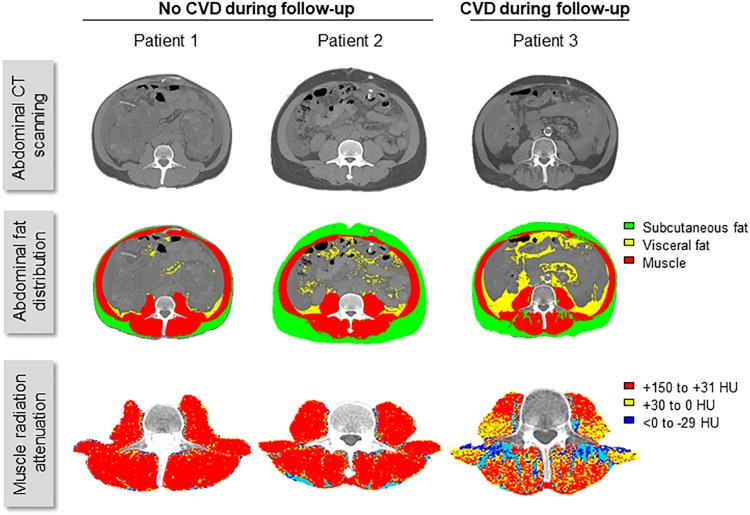
Fat distribution and the risk of CVD events in patients with kidney failure. Representative images of CT scan of the abdomen and analysis of fat distribution and muscle radiation attenuation (MRA) obtained at dialysis initiation from three male patients, matched for age, body-mass index, and residual urine volume. Age at CT scan was 52, 49, and 52-year-old; and body-mass index, 20.0, 21.4, and 19.4 kg/m^2^, for patients 1, 2, and 3, respectively. Urine output was > 1000 ml/day in all three patients. Despite similar clinical characteristics, mean MRA was 50.4, 50.9, and 22.7 HU, in patients 1, 2, and 3, respectively. Patient 3 presented cardiovascular disease (CVD) events during follow-up, in contrast to patients 1 and 2, who did not.

Patients with CVD events during follow-up were significantly older; had a higher comorbidity index, and higher plasma levels of C-reactive protein; they were more likely to have hypertension, diabetes, or a history of coronary heart disease, than those who did not develop CVD (Table 1). Patients with CVD during follow-up also had lower baseline values of MRA (reflecting excessive fat deposition in muscle) than patients without CVD (31 ± 11 *vs.* 38 ± 12 HU; *P* = 0.006) (Figure 1).

### Fat Accumulation in Skeletal Muscle Is Independently Associated With CVD in Kidney Failure

Based on the strong association between MRA and CVD observed in non-CKD individuals (Brumbaugh et al., 2012; Therkelsen et al., 2013; Lim and Meigs, 2014; Miljkovic et al., 2015; Zhao et al., 2016; Sugai et al., 2018), we tested whether baseline MRA reliably identifies patients with kidney failure at risk for subsequent CVD.

Survival analyses using Cox regressions identified higher baseline MRA (reflecting lower amounts of fat in skeletal muscle) as a strong negative predictor of CVD events during follow-up (unadjusted hazard ratio, 0.94; 95% confidence interval, 0.91–0.98, *P* = 0.004) (Table 2). The association was hardly changed after adjustment for additional risk factors (adjusted hazard ratio, 0.91; 95% confidence interval, 0.86–0.97, *P* = 0.006), or in sensitivity analyses (Table 2 and Supplementary Tables S3, S4). The use of a competing risk survival model considering transfer to hemodialysis and transplantation confirmed the association between low values of MRA at baseline and an increased hazard of CVD in the dialysis population (competing risks hazard ratio, 0.95; 95% confidence interval, 0.92–0.99, *P* = 0.007).

**TABLE 2:** Cox proportional hazard ratios for the time to first CVD event based on traditional risk factors and MRA at dialysis start.

	Unadjusted			Adjusted*		
		
	HR	95% CI	*P-*value	HR	95% CI	*P*-value
Age—years	1.03	1.01, 1.05	0.008	0.99	0.95, 1.03	0.6
HDL-cholesterol—mg/dL	0.99	0.97, 1.01	0.2	0.98	0.95, 1.01	0.2
Hypertension	4.77	1.14, 19.94	0.03	7.46	1.36, 40.87	0.02
Diabetes	3.02	1.53, 5.99	0.002	2.31	0.85, 6.27	0.1
CHD history	2.79	1.21, 6.42	0.02	2.01	0.61, 6.59	0.3
Body-mass index—kg/m^2^	1.09	1.02, 1.15	0.005	0.90	0.79, 1.02	0.09
Urine volume—mL	1.00	1.00, 1.00	0.9	1.00	1.00, 1.00	0.05
Plasma hsCRP—mg/dL	1.12	1.05, 1.19	<0.001	1.18	1.06, 1.32	0.003
MRA—HU	0.94	0.91, 0.98	0.004	0.91	0.86, 0.97	0.006

*Adjusted HR was calculated by integrating age, HDL-cholesterol, hypertension, diabetes, CHD history, body-mass index, residual urine volume, plasma hsCRP, and MRA. Log likelihood = -70.66. HR, hazard ratio; CI, confidence interval; CVD, cardiovascular disease; HDL, high density lipoprotein; CHD, coronary heart disease; hsCRP, high-sensitivity C-reactive protein; MRA, muscle radiation attenuation; HU, Hounsfield units.

Thus, low values of MRA at dialysis initiation represent a valuable and non-invasive indicator of increased risk of CVD in patients with kidney failure, independently of traditional risk factors.

### Fat Deposits Accumulate in Muscle During Time on Dialysis, and Are Independently Associated With Low Residual Urine Volume

**FIGURE 2:**
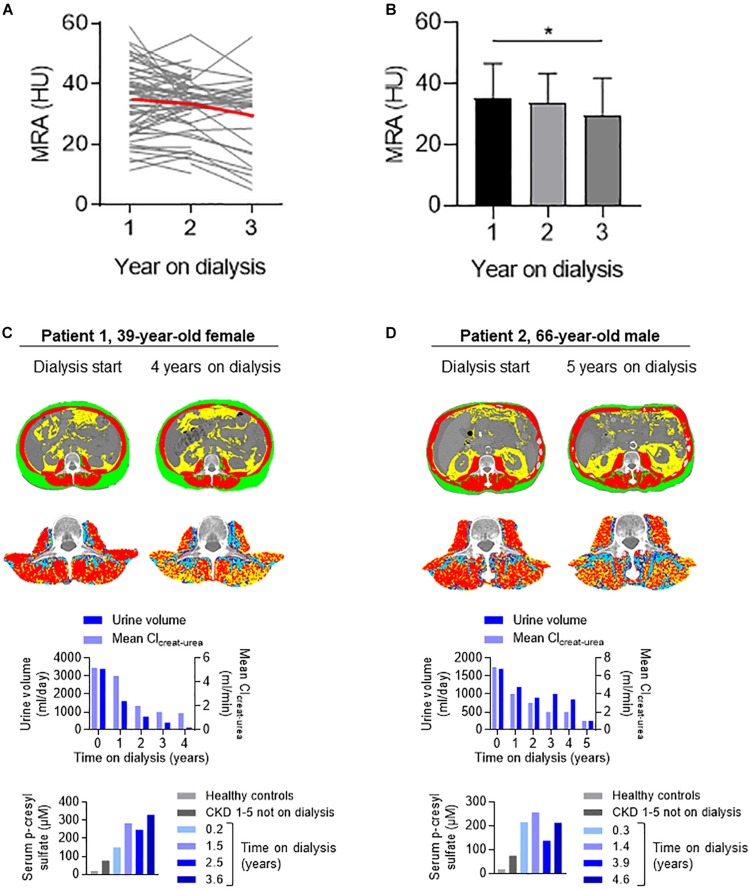
Longitudinal changes in muscle radiation attenuation during time on dialysis. **(A)** Individual changes (gray thin lines) and mean values (red thick line) of MRA during time on dialysis. **(B)** Mean (± SD) values of MRA during the first, second, and third years on dialysis (*P = 0.03 for linear trend, one-way ANOVA). **(C,D)** Illustrative longitudinal data of fat distribution, residual kidney function, and serum p-cresyl sulfate levels from two individuals on long-term PD: a 39-year-old woman on PD for 4 years (patient 1) and a 66-year-old man on PD for 5 years (patient 2). In both patients, a significant decrease in mean MRA (from 37.0 to 24.0 HU in patient 1 and from 37.6 to 27.2 HU in patient 2) paralleled the loss in residual function and accumulation of circulating p-cresyl sulfate.

To assess the dynamic changes in MRA over time on dialysis, we took advantage of the availability of a longitudinal follow-up of fat distribution, with CT scans performed yearly during time on PD. Considering repeated measures, we observed a significant decline in MRA over time, with a 6 and 17% decrease *vs.* baseline, at 12 and 24 months, respectively (Figures 2A,B and Supplementary Tables S1, S5). Other parameters of fat distribution were not influenced by time on dialysis (Supplementary Tables S1, S5).

Importantly, the association between MRA and time on dialysis attenuated and even disappeared after adjustment for residual urine volume, identified as an independent determinant of MRA in ESKD (Table 3). Other factors independently associated with MRA included older age, female gender, and VFA. On the contrary, the degree of glucose and icodextrin exposure—a reliable estimate of carbohydrates absorbed into the organism during PD, peritoneal solute transport rate (estimated from the dialysate-over-plasma ratio of creatinine) and net ultrafiltration were not associated with MRA.

**TABLE 3:** Determinants of MRA in patients with kidney failure treated with PD.

	Univariate analysis			Multivariate analysis		
		
	Coeff.	95% CI	*P*-value	Coeff.	95% CI	*P*-value
Age—years	–0.36	−0.46, −0.26	<0.001	–0.20	−0.34, −0.06	0.004
Female gender	–4.86	−9.63, −0.08	0.05	–6.60	−10.20, −3.00	<0.001
Diabetes	–6.68	−11.66, −1.70	0.009	3.14	−1.26,7.54	0.2
Charlson comorbidity index	–2.09	−2.81, −1.36	<0.001	–0.69	−1.56,0.19	0.1
Visceral fat area—cm^2^	–0.05	−0.07, −0.03	<0.001	–0.05	−0.07, −0.02	<0.001
Serum albumin g/L	0.31	0.03,0.59	0.03	0.24	−0.02,0.50	0.07
Urine volume—mL	2.66×10^−3^	0.91×10,-34.42×10-3	0.003	2.23×10^−3^	0.33×10,-34.13×10-3	0.02
Time on dialysis						
Year 1	1.00(*ref*.)	−	−	1.00(*ref*.)	−	−
Year 2	–1.84	−4.29,0.61	0.1	0.81	−1.24,2.85	0.4
Year 3	–4.32	−7.13, −1.51	0.003	0.22	−2.82,3.26	0.9
Year 4	–3.05	−6.08, −0.02	0.05	–0.27	−3.43,2.88	0.9
Constant				45.39	33.24,57.24	<0.001

*Coeff., coefficient; CI, confidence interval.*

Dynamic changes in MRA over time on dialysis and their association with low residual urine volume were illustrated by detailed monitoring of individuals on long-term dialysis. In these patients, a striking decrease in MRA paralleled the progressive decline in kidney function and the accumulation of p-cresyl sulfate, a uremic retention solute mechanistically involved in fat redistribution in experimental models of CKD (Figures 2C,D) (Koppe et al., 2013).

These data identified residual urine volume, age, gender, and VFA as independent determinants of MRA in patients with kidney failure. Longitudinal follow-up suggested MRA progressively decreases (fat accumulates in skeletal muscle) in parallel to declining residual urine volume in long-term PD patients.

### Kidney Failure Is Reversibly Associated With Fat Accumulation Skeletal Muscle

**FIGURE 3:**
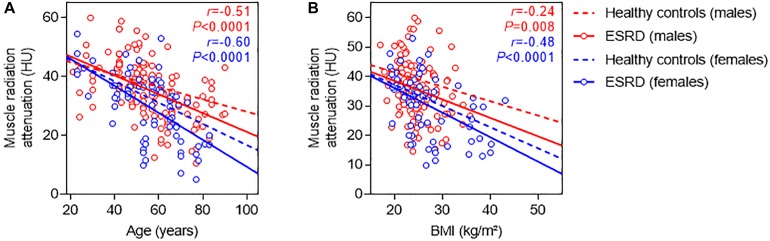
Relationship between muscle radiation attenuation, gender, age, and body-mass index in patients with kidney failure, and comparison with patients with normal kidney function. Relationship between MRA and age **(A)**, and MRA and body-mass index (BMI) **(B)**, among male (red) and female (blue) patients with ESKD. Regression lines between MRA and age, and MRA and BMI in patients with ESKD (continuous lines) are compared with those from reference values obtained in patients with normal kidney function (dotted lines) (van der Werf et al., 2018).

Based on these observations, we compared values of MRA in our cohort of incident patients with kidney failure to previously reported reference values from healthy controls with normal kidney function. Irrespective of gender, age, and body-mass index, patients with ESKD consistently had lower MRA than healthy controls, supporting the hypothesis that severely altered kidney function associates with fat accumulation in skeletal muscle (Figure 3).

**FIGURE 4:**
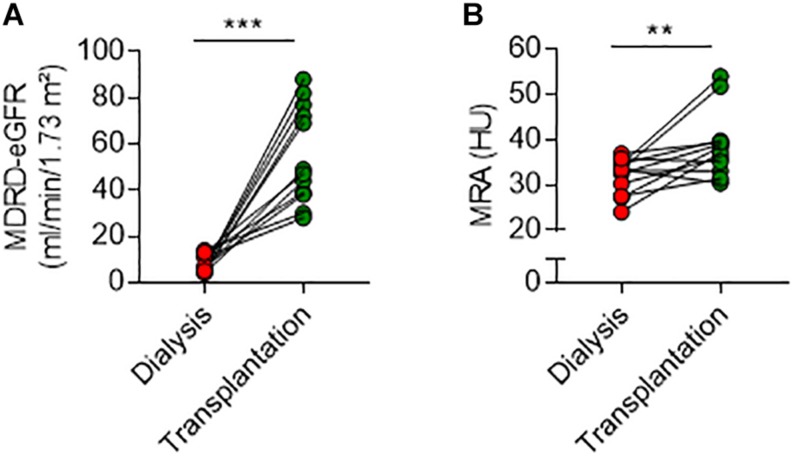
Dynamic changes in muscle radiation attenuation after kidney transplantation. Changes in MDRD-estimated glomerular filtration rate (eGFR) **(A)** and MRA **(B)** after kidney transplantation. Mean (±SD) MRA increased from 32.0 (±3.9) to 38.8 (±7.3) HU after transplantation (median of differences 5.41, Wilcoxon matched-pairs signed rank test, exact *P* = 0.009). **P < 0.01 and ***P < 0.001.

Next, we examined the longitudinal changes in MRA after transplantation and restoration of kidney function. We screened medical records and identified 12 patients from this cohort (five females/seven males, mean age ± SD, 51.3 ± 14.5 years) who received a kidney transplant after dialysis, and in whom an abdominal non-contrast enhanced CT scanning was also performed after transplantation for an unrelated clinical indication. All patients received a standard immunosuppression regimen containing mycophenolate mofetil, tacrolimus, and rapidly tapered doses of corticosteroids. After a median time [IQR] from transplantation to CT scanning of 169 days [82–803], MDRD-estimated glomerular filtration rate had increased from 6 [5–12] to 55 [38–76] ml/min/1.73 m^2^ (*P* < 0.001) (Figure 4). In parallel, MRA increased in 9/12 (75%) patients, remained stable in 2/12 (17%), and slightly decreased in 1/12 (8%) (Figure 4). Overall, mean (±SD) MRA increased after transplantation from 32.0 (±3.9) to 38.8 (±7.3) HU (median of differences 5.41, exact *P* = 0.009).

Altogether, these data showed in a small number of patients that kidney transplantation is associated with a rapid increase in MRA, supporting the hypothesis that kidney failure contributes to lipid redistribution and ectopic fat deposition in skeletal muscle.

## Discussion

In a cohort of incident ESKD patients, our study shows, for the first time, a strong and independent association between low values of MRA—a surrogate for ectopic fat accumulation in skeletal muscle—and an increased risk for CVD. A low MRA at dialysis initiation is a reliable predictor of CVD, independently of traditional risk factors, suggesting its potential to identify high risk ESKD patients. Several observations also suggest that ESKD may promote ectopic fat redistribution, which in turn could be reversed by restoration of kidney function.

The worldwide obesity epidemic has emerged as a major cause of insulin resistance and type 2 diabetes, and such metabolic alterations now represent the leading risk factors for CVD. Classically defined by body-mass index, overweight and obesity are heterogeneous conditions, with important inter-individual differences in risk factor profiles despite similar amounts of total body fat, including in the CKD population (Stenvinkel et al., 2013). Anthropometric measures such as waist circumference and waist-to-hip ratio and, more recently, novel imaging techniques contributed to demonstrate the critical role of regional fat distribution in the development of metabolic alterations and CVD (Pou et al., 2007; Liu et al., 2010, 2011; Therkelsen et al., 2013; Yoshitaka et al., 2017; Neeland et al., 2018, 2019; Okamura et al., 2019; Tanaka et al., 2019). In the present work, ectopic fat in skeletal muscle was strongly associated with CVD, extending for the first time the key role of altered fat distribution in the pathophysiology of CVD to the population with ESKD. In this cohort, low values of MRA at dialysis initiation identified patients at risk for subsequent CVD events, and every one HU decrease in MRA was associated with a ∼5–10% increase in the probability of CVD.

The mechanisms by which ectopic fat accumulation promotes insulin resistance and CVD have only been partially elucidated (Shulman, 2014; Samuel and Shulman, 2016; Neeland et al., 2018; Petersen and Shulman, 2018). An accumulating body of evidence suggests that intramyocellular accumulation of lipid metabolites directly leads to insulin resistance, *via* defects in insulin signaling and reduced insulin-stimulated glucose-transport activity (Shulman, 2014; Samuel and Shulman, 2016; Petersen and Shulman, 2018). Additional mechanisms underlying the association between fatty muscle infiltration and CVD have been suggested, including the release of proinflammatory adipokines from adipocytes surrounding muscle fibers; altered mitochondrial function and increased oxidative stress in skeletal muscle cells; and accelerated aging (Pou et al., 2007; Miljkovic et al., 2015). At the systemic level, ectopic adiposity also increases circulating blood volume and pro-atherogenic inflammatory factors, contributing to cardiac wall stress, myocardial injury, and left ventricle hypertrophy and remodeling (Neeland et al., 2018). As fatty muscle infiltration parallels ectopic fat accumulation in the organism, it is tempting to speculate that at least part of the association with cardiometabolic risk is mediated by fat accumulation in other organs, such as the liver or the visceral adipose tissue (Stefan et al., 2013; Artunc et al., 2016). In particular, lipid accumulation in the liver (non-alcoholic fatty liver disease) has been shown to promote hepatic glucose production, insulin resistance, dyslipidemia, and dysregulated hepatokine production (Stefan et al., 2019).

These data also suggest that impaired kidney function is linked to ectopic fat accumulation in skeletal muscle. First, in the whole cohort, MRA strongly and independently associated with residual urine volume. Second, longitudinal monitoring of patients on dialysis showed a progressive accumulation of lipids in skeletal muscle, which paralleled kidney function decline and accumulation of uremic retention solutes. Third, as compared with a control population with normal kidney function (Goodpaster et al., 2000a; van der Werf et al., 2018), MRA was consistently lower among patients on dialysis, irrespective of age, gender, and body fat distribution. Lastly, successful kidney transplantation was followed by a significant increase in MRA, suggesting the reversibility of ectopic lipid accumulation in skeletal muscle after restoration of kidney function. To the best of our knowledge, these observations are the first to suggest that kidney disease *per se* is associated with ectopic fat accumulation in human skeletal muscle, independently of other covariates, including central adiposity.

These data are in line with experimental studies in rodents, in which nephron mass reduction leads to a reduction of fat depot size and ectopic lipid redistribution (Zhao et al., 2008; Koppe et al., 2013; Pelletier et al., 2013). The mechanisms by which CKD promotes ectopic fat and insulin resistance are likely multifactorial, potentially including alterations of gut microbiota, adipokines dysregulations, inflammation and oxidative stress in adipocyte cells, and increased lipolysis (Koppe et al., 2014). Recent evidence demonstrated a central role for p-cresyl sulfate, a gut-derived protein-bound uremic retention solute cleared by the kidneys and poorly dialyzed by conventional techniques. Long-term administration of p-cresyl sulfate to mice was sufficient to induce the same degree of lipid redistribution and insulin resistance as did severe CKD (Koppe et al., 2013). In these models, treatment with a prebiotic reducing intestinal production of p-cresyl sulfate prevented the accumulation of ectopic lipids in muscle and improved CKD-associated insulin resistance (Koppe et al., 2013). In individuals on long-term PD, loss of kidney function was associated with increasing levels of uremic retention solutes, with 4- and 15-fold increases in circulating levels of total p-cresyl sulfate as compared with CKD or healthy controls, respectively. Future trials will need to test whether enhanced removal of protein-bound uremic toxins (e.g., using intensive dialysis regimens or convective therapies), prebiotics, or other pharmacological approaches efficiently reduce ectopic fat accumulation in patients with ESKD, and whether these strategies mitigate the unacceptably high risk of CVD in this population.

This original study has several strengths, including the use of the gold standard method for the evaluation of fat distribution; systematic longitudinal monitoring with non-contrast enhanced CT scans; and detailed follow-up of global and CVD outcomes in a high-risk population of patients with ESKD. The use of a technique based on the universal and highly standardized HU scale for the measurement of MRA ensures reproducibility and allows direct comparison with existing cohorts. We also acknowledge limitations, including the absence of evaluation of insulin resistance; muscle strength; hydration status; and ectopic fat accumulation in other locations, including the liver and cardiomyocytes. As with all dialysis studies, informative censoring is a potential issue. To limit potential bias due to non-random dropout of patients, we adjusted time-to-event analyses for dialysis duration and outcome after PD, and also applied a competing risk model. Concerns about the long-term consequences of repeat radiation exposure represent a potential limitation to the clinical application of CT-based techniques to assess fat distribution (Brenner and Hall, 2007). However, the use of single slice abdominal CT at the level of the third lumbar vertebra reduces radiation dose to less than 1 mSv (unpublished data), which is at least 10 times smaller than the corresponding dose for a whole abdomen CT scan, therefore opening translational perspectives.

## Conclusion

In summary, we demonstrated in a cohort of incident patients with kidney failure that MRA is associated with CVD, independently of traditional risk factors. The use of a robust and reliable technique, based on non-contrast CT scanning of the abdomen, successfully identified incident ESKD patients at risk for subsequent CVD. Our data also suggest a close relationship between CKD and ectopic fat redistribution, a finding that may potentially explain the unacceptably high burden of CVD among patients with CKD.

## Data Availability Statement

The datasets generated during the current study are available from the corresponding author on reasonable request.

## Ethics Statement

The study was in accordance with the World Medical Association’s Declaration of Helsinki, the Belgian law related to experiments in humans dated 7 May 2004, the General Data Protection Regulation 2016/679, and the Belgian law of 30 July 2018 regarding the protection of personal data. The Ethical Review Board of Cliniques universitaires Saint-Luc and UCLouvain approved the study (approval number: 2015/10DEC/675) and waived the requirement to obtain informed consent based on the observational and retrospective design of the study.

## Author Contributions

MK, TM, and JM contributed to research idea, study design, and drafting of the manuscript. MK, TM, EC, PT, MN, RC, CC, BB, and JM contributed to data acquisition, analysis, and interpretation. EC, RC, and JM contributed to statistical analysis. EC, MN, RC, CC, BB, MJ, and EG contributed to critical revision of the manuscript for important intellectual content. RC, MJ, EG, and JM contributed to study supervision or mentorship. Each author contributed important intellectual content during manuscript drafting or revision and accepts accountability for the overall work by ensuring that questions pertaining to the accuracy or integrity of any portion of the work are appropriately investigated and resolved.

## Conflict of Interest

The authors declare that the research was conducted in the absence of any commercial or financial relationships that could be construed as a potential conflict of interest.
