# Supplementary material for: Non-invasive Quantification of Fat Deposits in Skeletal Muscle Predicts Cardiovascular Outcome in Kidney Failure
Source: Front Physiol. 2020 Feb 25;11:130. doi: 10.3389/fphys.2020.00130 (PMC7053428; doi:10.3389/fphys.2020.00130)
Supplement: Supplementary file 1 [file Table_1.DOCX]

***Supplementary Material***

***Table of Content***

**Suppl. Table 1.** Fat distribution at dialysis initiation and during treatment

**Suppl. Table 2.** Outcome and CVD events on dialysis

**Suppl. Table 3.** Sensitivity analyses for the time to first CVD event based on risk factors at dialysis start, taking into account gender and medication use

**Suppl. Table 4.** Sensitivity analyses for the time to first CVD event based on risk factors at dialysis start, after exclusion of the variable age at dialysis start

**Suppl. Table 5.** Changes in fat distribution during time on dialysis

This supplementary material has been provided by the authors to give readers additional information about their work.

**Suppl. Table 1.** Fat distribution at dialysis initiation and during treatment.

|  | **Year 1** | **Year 2** | **Year 3** |
| --- | --- | --- | --- |
| Subcutaneous fat area – cm² | 143±88 | 153±101 | 168±105 |
| Visceral fat area – cm² | 118±86 | 132±101 | 142±85 |
| Skeletal muscle index – cm² | 51±9 | 52±10 | 50±9 |
| Muscle radiation attenuation – HU | 36±12 | 34±10 | 30±12 |

Results are presented as mean ± SD; HU, Hounsfield Units.

**Suppl. Table 2.** Outcome and CVD events on dialysis.

|  | **Value** |
| --- | --- |
| Median follow-up – mo. | 21 [9-40] |
| Outcome |  |
| Kidney transplantation – n (%) | 31 (31) |
| Transfer to hemodialysis – n (%) | 31 (31) |
| Death – n (%) | 22 (22) |
| Still on PD at last follow-up – n (%) | 12 (12) |
| Recovery or lost to follow-up – n (%) | 5 (5) |
| No. of non-fatal CVD events | 58 |
| Number of patients with ≥1 non-fatal CVD event– n (%) | 34 (34) |
| Time to first non-fatal CVD event – mo. | 15 [5-24] |
| Type of non-fatal CVD event – n (%) |  |
| Myocardial infarction or revascularization | 17 (29) |
| Stroke or transient ischemic attack | 12 (21) |
| Lower limb necrosis or revascularization | 29 (50) |

Results are presented as median [IQR] or number (n) and percentage (%) as appropriate. PD, peritoneal dialysis; CVD, cardiovascular disease.

**Suppl. Table 3.** Sensitivity analyses for the time to first CVD event based on risk factors at dialysis start, taking into account gender and medication use.

|  | | | | | | |  |
| --- | --- | --- | --- | --- | --- | --- | --- |
|  | **Unadjusted** | | | **Adjusted*** | | | |
|  | **HR** | **95% CI** | ***P* Value** | **HR** | **95% CI** | ***P* Value** | |
| Age - years | 1.03 | 1.01, 1.05 | 0.008 | 0.99 | 0.94, 1.04 | 0.6 | |
| Gender | 1.00 | 0.50, 2.01 | 0.9 | 0.61 | 0.18, 2.13 | 0.4 | |
| HDL-cholesterol – mg/dL | 0.99 | 0.97, 1.01 | 0.2 | 0.99 | 0.95, 1.02 | 0.4 | |
| Hypertension | 4.77 | 1.14, 19.94 | 0.03 | 3.96 | 0.68, 23.16 | 0.1 | |
| Diabetes | 3.02 | 1.53, 5.99 | 0.002 | 1.83 | 0.47, 7.11 | 0.4 | |
| CHD history | 2.79 | 1.21, 6.42 | 0.02 | 2.95 | 0.59, 14.83 | 0.2 | |
| Body mass index – kg/m^2^ | 1.09 | 1.02, 1.15 | 0.005 | 0.89 | 0.77, 1.02 | 0.09 | |
| Resid. urine volume - mL | 1.00 | 1.00, 1.00 | 0.9 | 1.00 | 1.00, 1.00 | 0.03 | |
| Plasma hsCRP –mg/dL | 1.12 | 1.05, 1.19 | <0.001 | 1.17 | 1.03, 1.33 | 0.01 | |
| Statin use | 2.00 | 0.93, 4.29 | 0.08 | 0.60 | 0.18, 1.94 | 0.4 | |
| ACE inhibitor use | 1.91 | 0.95, 3.82 | 0.07 | 3.23 | 0.95, 10.96 | 0.06 | |
| ARB use | 0.94 | 0.47, 1.88 | 0.9 | 1.73 | 0.54, 5.52 | 0.4 | |
| Beta-blocker use | 1.52 | 0.77, 2.98 | 0.2 | 0.54 | 0.17, 1.66 | 0.3 | |
| Insulin use | 5.11 | 2.43, 10.77 | <0.001 | 1.78 | 0.28, 11.49 | 0.6 | |
| MRA - HU | 0.94 | 0.91, 0.98 | 0.004 | 0.91 | 0.86, 0.97 | 0.006 | |

*Adjusted HR were calculated by integrating age, gender, HDL-cholesterol, hypertension, diabetes, CHD history, body-mass index, residual urine volume, plasma hsCRP, use of statins, angiotensin converting enzyme inhibitors or angiotensin receptor blockers, insulin, beta-blockers, and MRA. Log likelihood = -66.70. HR, hazard ratio; CI, confidence interval; CVD, cardiovascular disease; HDL, high density lipoprotein; CHD, coronary heart disease; hsCRP, high-sensitivity C-reactive protein; MRA, muscle radiation attenuation; HU, Hounsfield units.

**Suppl. Table 4.** Sensitivity analyses for the time to first CVD event based on risk factors at dialysis start, after exclusion of the variable age at dialysis start.

|  | | | | | | |
| --- | --- | --- | --- | --- | --- | --- |
|  | **Unadjusted** | | | **Adjusted*** | | |
|  | **HR** | **95% CI** | ***P* Value** | **HR** | **95% CI** | ***P* Value** |
| HDL-cholesterol – mg/dL | 0.99 | 0.97, 1.01 | 0.2 | 0.98 | 0.95, 1.01 | 0.2 |
| Hypertension | 4.77 | 1.14, 19.94 | 0.03 | 7.02 | 1.31, 37.62 | 0.02 |
| Diabetes | 3.02 | 1.53, 5.99 | 0.002 | 2.24 | 0.83, 6.04 | 0.1 |
| CHD history | 2.79 | 1.21, 6.42 | 0.02 | 1.86 | 0.60, 5.83 | 0.3 |
| Body mass index – kg/m^2^ | 1.09 | 1.02, 1.15 | 0.005 | 0.91 | 0.82, 1.02 | 0.1 |
| Resid. urine volume - mL | 1.00 | 1.00, 1.00 | 0.9 | 1.00 | 1.00, 1.00 | 0.05 |
| Plasma hsCRP –mg/dL | 1.12 | 1.05, 1.19 | <0.001 | 1.17 | 1.06, 1.29 | 0.002 |
| MRA - HU | 0.94 | 0.91, 0.98 | 0.004 | 0.92 | 0.87, 0.97 | 0.002 |

*Adjusted HR were calculated by integrating HDL-cholesterol, hypertension, diabetes, CHD history, body-mass index, residual urine volume, plasma hsCRP, and MRA. Log likelihood = -70.78. HR, hazard ratio; CI, confidence interval; CVD, cardiovascular disease; HDL, high density lipoprotein; CHD, coronary heart disease; hsCRP, high-sensitivity C-reactive protein; MRA, muscle radiation attenuation; HU, Hounsfield units.

**Suppl. Table 5.** Changes in fat distribution during time on dialysis.

| **Time on PD (yr)** | **Subcutaneous fat area** | | | **Visceral fat area** | | | **Skeletal muscle index** | | | **Muscle radiation attenuation** | | |
| --- | --- | --- | --- | --- | --- | --- | --- | --- | --- | --- | --- | --- |
|  | **Coeff.** | **95% CI** | ***P* Value** | **Coeff.** | **95% CI** | ***P* Value** | **Coeff.** | **95% CI** | ***P* Value** | **Coeff.** | **95% CI** | ***P* Value** |
| 1 | 5.65 | -5.48, 16.79 | 0.3 | 4.79 | -7.51, 17.09 | 0.5 | 1.17 | -0.24, 2.58 | 0.1 | -1.82 | -4.29, 0.65 | 0.2 |
| 2 | -0.76 | -18.04, 16.53 | 0.9 | -2.10 | -19.61, 15.42 | 0.8 | -1.00 | -2.92, 0.92 | 0.3 | -4.30 | -7.12, -1.48 | 0.003 |
| 3 | 7.40 | -10.22, 25.02 | 0.4 | 6.61 | -13.55, 26.77 | 0.5 | 0.95 | -0.46, 2.35 | 0.2 | -3.03 | -6.06, -0.01 | 0.05 |
| 4 | -19.02 | -46.76, 8.72 | 0.2 | -12.32 | -30.43, 5.79 | 0.2 | -0.70 | -3.28, 1.88 | 0.6 | -6.39 | -8.76, -4.01 | <0.001 |

Coeff., coefficient; CI, confidence interval; PD, peritoneal dialysis; yr, year.
